# Photocatalytic Hydrogen Production and Carbon Dioxide Reduction Catalyzed by an Artificial Cobalt Hemoprotein

**DOI:** 10.3390/ijms232314640

**Published:** 2022-11-24

**Authors:** Guillermo A. Oliveira Udry, Laura Tiessler-Sala, Eva Pugliese, Agathe Urvoas, Zakaria Halime, Jean-Didier Maréchal, Jean-Pierre Mahy, Rémy Ricoux

**Affiliations:** 1 UMR 8182, CNRS, Institut de Chimie Moleculaire & des Matériaux d’Orsay, University Paris-Saclay, F-91405 Orsay, France; 2 Departament de Química, Universitat Autònoma de Barcelona, 08193 Bellaterra, Spain; 3 CEA, CNRS, Institute for Integrative Biology of the Cell (I2BC), University Paris-Saclay, F-91198 Gif-sur-Yvette, France

**Keywords:** artificial metalloenzymes, cobalt porphyrin, proton reduction, CO_2_ reduction, catalysis

## Abstract

The covalent insertion of a cobalt heme into the cavity of an artificial protein named alpha Rep (αRep) leads to an artificial cobalt hemoprotein that is active as a catalyst not only for the photo-induced production of H_2_, but also for the reduction of CO_2_ in a neutral aqueous solution. This new artificial metalloenzyme has been purified and characterized by Matrix Assisted Laser Desorption Ionization-Time of Flight Mass Spectrometry (MALDI-TOF MS), circular dichroism, and UltraViolet–Visible spectroscopy. Using theoretical experiments, the structure of this biohybrid and the positioning of the residues near the metal complex were examined, which made it possible to complete the coordination of the cobalt ion by an axial glutamine Gln283 ligand. While the Co(III)–porphyrin catalyst alone showed weak catalytic activity for both reactions, 10 times more H_2_ and four times more CO_2_ were produced when the Co(III)–porphyrin complex was buried in the hydrophobic cavity of the protein. This study thus provides a solid basis for further improvement of these biohybrids using well-designed modifications of the second and outer coordination sphere by site-directed mutagenesis of the host protein.

## 1. Introduction

One of the greatest challenges facing our society is to replace current fuels with new, abundant, and renewable sources of energy. Hydrogen (H_2_) seems to be the ideal energy carrier because, in addition to its highly energetic H–H bond, its environmentally friendly combustion releases only water as a product. H_2_ production processes are numerous (thermal, photochemical, biological, electrolytic, etc.) and the resources used in these processes are equally as abundant (water, solid waste, cellulose, biomass, etc.). To meet the fast-growing global demand for H_2_, the development of catalysts for the production of hydrogen is highly sought after. Specifically, new original molecular complexes, most often bioinspired, based on non-noble metals such as nickel, cobalt, or molybdenum have been synthesized and studied to replace platinum, a catalyst that is certainly effective but scarce and expensive [1,2,3,4]. Molecular catalysts have been shown to be proficient in the hydrogen evolution reaction (HER), showing increased efficiency and stability. In particular, a variety of molecular cobalt catalysts have been developed for the production of hydrogen such as glyoxime and the vitamin B12, which have the disadvantage of being poorly soluble and unstable in water [5]. These include mononuclear and dinuclear cobalt polypyridines [6,7]; macrocyclic ligands such cobalt corroles [8]; cationic cobalt porphyrins [9]; anionic cobalt porphyrins [10]; cobalt heme-peptides [11]; and more recently, a synthetic mini-enzyme known as mimochrome VI*a [12]. These cobalt complexes underwent a light-induced proton reduction in water when combined with a photosensitizer and a sacrificial electron donor. However, many of these complexes are sparingly soluble in water and their chemical stability is not optimal. To improve the catalytic performance of these systems, their outer coordination sphere can be designed to promote the efficient transfer of electrons, H^+^ or H_2_. To overcome this limitation, efforts have been made to develop artificial metalloenzymes with an unnatural active site integrated into a protein. This strategy has been shown to improve the production of H_2_ [13] and different artificial Co-artificial metalloenzymes have been obtained by non-covalent insertion of cobaloxime derivatives into apo-hemoproteins such as sperm whale myoglobin or heme oxygenase [14,15] or in a protein carrying out electron transfers such as ferredoxin [16]. In order to improve the catalytic efficiency of those artificial metalloenzymes, another cofactor such as the cobalt protoporphyrin IX was incorporated into a sperm whale myoglobin scaffold by the group of Ghirlanda [17]. In a recent publication, the same group successfully reported the light-driven reduction of CO_2_ and the generation of H_2_ using an artificial hemoprotein made from the Cytochrome b562 apoprotein that included Co-Protoporphyrin IX as a cofactor [18]. Inspired by these results, we decided to assemble new artificial metalloenzymes by a covalent-grafting strategy, involving the synthesis of various cobalt cofactors bearing a moiety that would be able to react with the SH group of a cysteinyl residue that could permanently graft them to the protein. Recently, our group has shown that a new class of thermostable artificial proteins named αReps have the potential to be transformed into artificial metalloenzymes by covalent grafting of a synthetic metal complex [19]. These αRep proteins are easily produced in *E. coli*, are very stable within a large pH range, and exhibit a large hydrophobic core. We have thus previously reported a proof of concept by creating artificial Diels–Alderases by covalent attachment of phenanthroline– or terpyridine–Cu(II) complexes in a specific αRep single-chain asymmetric bidomain variant named (A3A3′)Y26C [20]. This protein has been shown to adopt a closed bivalve shell-like conformation and molecular modeling identified that this cavity is large enough for the anchoring of even larger metal complexes such as *meso*-tetraphenylporphyrin [21]. Thus, from an enzyme designer’s point of view, the artificial αRep proteins bring together the properties required for the development of artificial metalloenzymes dedicated to H_2_ production. Accordingly, we here report the production and characterization of a new artificial metalloenzyme obtained by the covalent attachment of a cobalt porphyrin derivative into the αRep (A3A3′)Y26C variant and further shows its remarkable activity to catalyze the photoinduced production of H_2_ and CO_2_ reduction.

## 2. Results and Discussion

### 2.1. Synthesis of Co(III)Mal-PPIXMME ***4***

The Co(III)Mal-PPIXMME cofactor **4** was prepared in three steps. In the first step was the hydrolysis of protoporphyrin IX dimethyl ester (PPIXDME) by 6 N HCl for 15 min at room temperature (RT) following the reaction conditions described by Ellsworth R.K. [22] (see Materials and Methods). Protoporphyrin IX monomethyl ester (PPIXMME) **2** was then obtained in a 28% yield, and was characterized by NMR and UV–Visible spectroscopies as well as by ESI MS (see Materials and Methods).

**Figure 1:**
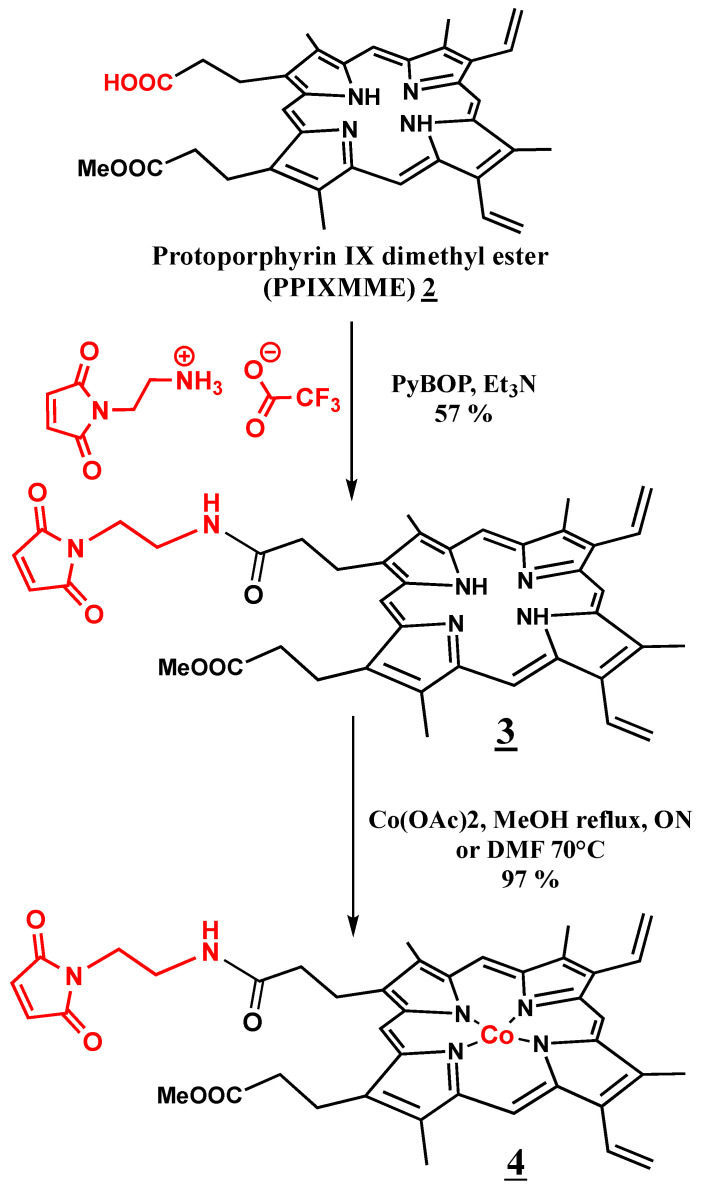
Synthesis of the Co(III)Mal-PPIXMME cofactor **4** from protoporphyrin IX monomethyl ester (PPIX MME) **1**.

The two following steps of the synthesis are shown in Figure 1. The reaction of **2** with a slight excess of *N*-(2-aminoethyl)-maleimide trifluoroacetate salt (1.1 equiv.) in the presence of triethylamine and PyBOP as a coupling agent in dimethylformamide (DMF) for 30 min at RT afforded the maleimido-ethylamido-protoporphyrin IX monomethyl ester Mal-PPIXMME **3** as a brown solid in a 45% yield. **3** was characterized by NMR and UV–Visible spectroscopies and ESI MS (see Materials and Methods). These characteristics were in good agreement with those of a very similar product, maleimido-ethylamido-protoporphyrin IX monoethyl ester Mal-PPIXMEE, which has already been reported by the group of Y. Watanabe [23].

Finally, the addition of 10 equiv. of Co(OAC)_2_, 4H_2_O to the obtained compound **3** in anhydrous DMF at 70 °C led, after the removal of the excess of cobalt salt by extraction in a dichloromethane (DCM)/water mixture and the removal of the solvent under reduced pressure, to Co(III)-Mal-PPIXMME as a dark green solid in a 97% yield. **4** was characterized by UV–Visible spectroscopy and ESI MS spectrometry.

### 2.2. Biohybrid Preparation

The (A3A3′)Y26C-Co(III)Mal-PPIXMME (A3A3′)Y26C-**4**) biohybrid was prepared in three steps. The single-chain bidomain protein in which a cysteine-free domain is linked with a domain bearing the Y26C mutation ((A3A3′)Y26C) was first produced and purified, as recently described [24]. Compound **4** was then synthesized from protoporphyrin IX in three steps, as described above. The biohybrid was finally assembled by incubation of the (A3A3′)Y26C protein with 10 eq. of compound **4** for 3 days at 4 °C in a 50 mM Na-phosphate pH 7.75 coupling buffer containing 150 mM NaCl and 25% dimethyl sulfoxide (DMSO). After removal of the excess of compound **4** by extraction with butan-2-one, the (A3A3′)Y26-**4** biohybrid was recovered in the aqueous phase. 

**Figure 2:**
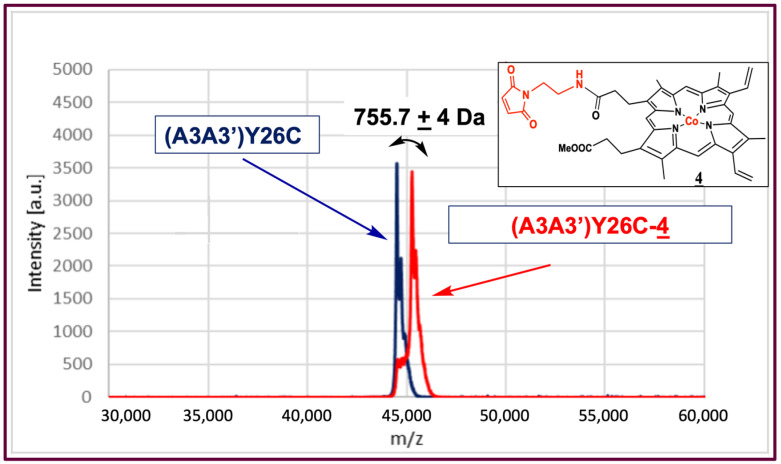
Mass spectrometry analysis of the (A3A3′)Y26C-**4** biohybrid: Maldi-ToF spectrum of (A3A3′)Y26C (blue) and (A3A3′)Y26C-**4** (red).

The (A3A3′)Y26C-**4** biohybrid was then characterized by MALDI-ToF mass spectrometry analysis (Figure 2) where (A3A3′)Y26C showed a major peak at M = 44,502 Da, and a peak at 45,262 Da was observed for the (A3A3′)Y26C-**4** biohybrid, which was in agreement with the covalent attachment of compound **4** (M = 755.7 Da) on (A3A3′)Y26C.

**Figure 3:**
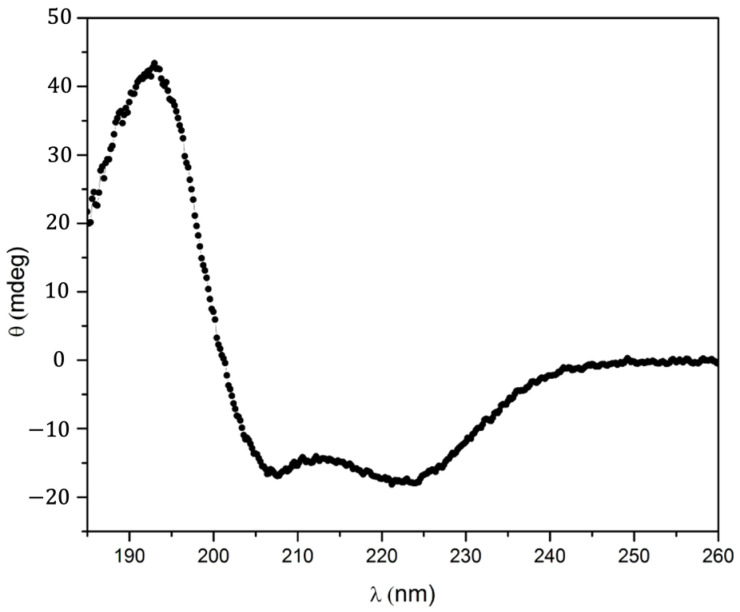
Far-UV CD spectrum of a 2.2 mM solution of the (A3A3′)Y26C-**4** biohybrid in 20 mM sodium phosphate buffer pH 7.5.

To analyze the effect of the binding of **4** into the cavity of (A3A3′)Y26C on the folding of the protein, circular dichroism (CD) measurements were carried out. The far UV circular dichroism spectrum of the (A3A3′)Y26C-**4** biohybrid (Figure 3) showed a signal with two negative bands at 222 and 208 nm and a positive band around 190 nm that was characteristic of proteins containing mainly α helices as a secondary structure. This signal was very similar to that of the non-modified (A3A3′)Y26C protein [19,21], indicating that its 3D structure was not altered after covalent attachment of the porphyrin cofactor in its hydrophobic pocket, even in the presence of 25% DMSO, and further treatment with butan-2-one.

### 2.3. UV–Visible Characteristics of the (A3A3′)Y26C-***4*** Biohybrid

**Figure 4:**
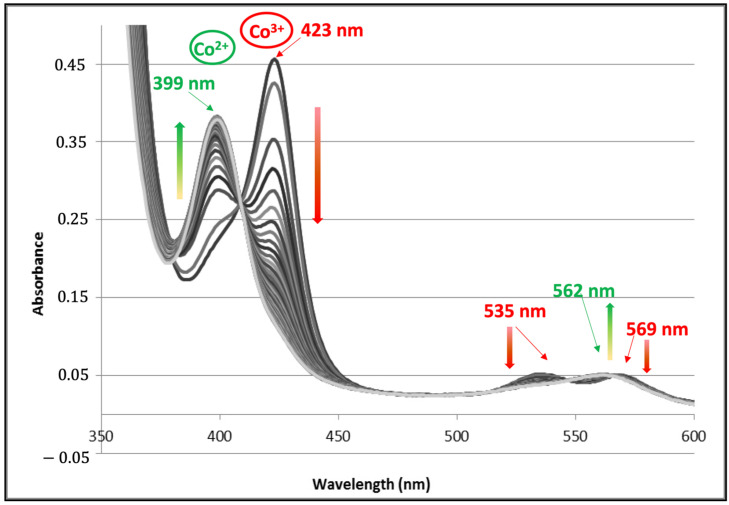
Evolution of the UV–Vis spectrum of (A3A3′)Y26C-**4** 7.5 µM in 10 mM HEPES, NaCl 150 mM buffer, pH 8.0 after the addition of a 1.5 mM anaerobic solution of sodium dithionite. Spectra were recorded every 5 min during 2 h.

The addition, under anaerobic conditions, of 1.5 mM sodium dithionite to a 7.5 µM solution of (A3A3′)Y26C-**4** in 10 mM HEPES, NaCl 150 mM buffer, pH 8.0 led to the gradual replacement, with an isosbestic point at 408 nm, of the spectrum characteristic of Co(III)-Mal-PPIXMME **4** with peaks at 423, 535, and 569 nm by a new one with peaks at 399 and 562 nm, which was characteristic of the reduced form of the biohybrid Co(II)–Mal-PPIXMME **4** (Figure 4).

The characteristics of these spectra are compared in Table 1 with those obtained after the incorporation of Co(III)– and Co(II)–protoporphyrin IX into the apoprotein of various hemoproteins including myoglobin (Mb) [24] and its H64V-V68A double mutant [25]; two cytochromes P450, P450_Cam_ [26] and CYP119 [27]; two peroxidases, horseradish peroxidase (HRP) [24] and dye decolorizing peroxidase (DYP) [28], aldoxime dehydrogenase (Oxd) [28] and catalase (Cat) [28].

As shown in Table 1, the electronic absorption spectrum of the oxidized Co(III)–(A3A3′)Y26C-**4** biohybrid was similar to those obtained through the incorporation of Co(III)PPIX not only into apoproteins, which bind the cobalt cation through an axial thiolate ligand such as P450 cam [26] and CYP 119 [27], but also to those of some apoproteins that bind the cobalt cation through an axial histidine ligand such as Mb, HRP [24], and DYP [28]. Such a comparison does not allow for the identification the cobalt axial ligand of cobalt in (A3A3′)Y26C-**4**. However, when comparing the electronic absorption spectra of the various reduced complexes, it appears that the spectral characteristics of the Co(II)–(A3A3′)Y26C-**4** biohybrid were very different from those observed with P450 cam [26] and CYP 119 [27] whereas they were closer than those observed in the case of proteins bearing an axial histidine ligand with, in particular, a very strong similarity to those of the Co(II) dye decolorizing peroxidase (DYP) [28]. This latter comparison tends to suggest axial coordination of the cobalt ion by a histidine ligand in the Co(II)–(A3A3′)Y26C-**4** biohybrid. However, in order to clarify the environment of the cobalt in the distal pocket of this artificial metalloenzyme, and in particular, the nature of its axial ligand, computational modeling studies were engaged.

### 2.4. Computational Modeling Studies

In the absence of the X-ray structure of the (A3A3′)Y26C-**4** biohybrid, we embarked on a molecular modeling study. It primarily aimed at elucidating the most stable orientation of the non-natural porphyrin into (A3A3′)Y26C and assessing the relevant interactions between both subsystems including possible coordination bonds between nearby lying amino acids and the metal.

First, protein–ligand dockings were carried out. For the protein structure, we used the representative geometry of the most populated cluster of conformations of a 300 ns molecular dynamics (MD) performed with AMBER [29] of the wild type A3A3′ for which Tyr26 was mutated to cysteine using the UCSF Chimera plug-in for amino acid substitution [30]. The artificial cofactor was accordingly docked using the covalent protocol available in Gold and the GoldScore scoring function that we updated for the metalloligand recently [31].

**Figure 5:**
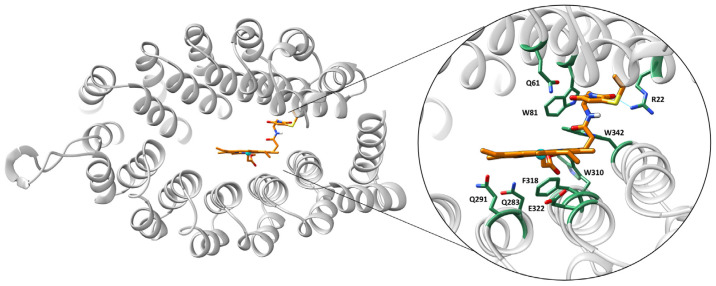
Representation of the lowest energy geometry of the (A3A3′)Y26C-**4** biohybrid obtained with Gold and the Goldscore scoring function.

A first docking run was performed with a rigid protein scaffold. From the 50 solutions obtained, the lowest energy solution reached 55.1 GoldScore units and can be considered to be good interaction between both molecular partners. The porphyrin stands very well into the inter-dominial pocket and displays good interactions with amino acids of the cavity such as Phe318 with the macrocycle, the methyl group of the methyl propionate ester side chain interacts with a hydrophobic patch constituted by Trp81, Trp310, and Trp342, and the cyclic moiety of the linker interacting polarity with Arg22 (Figure 5). Close-lying residues that could coordinate are Gln 61,283 and 291 or Asp322. 

Based on this structure, further dockings were carried out to see whether nearby lying residues could eventually interact with the metal. This was performed by giving rotational flexibility to the side chain of the amino acid Glu322 and Phe318 as well as Gln283. From those calculations, it appears that Glu322 and Gln291 may both reach convenient distances for coordination, though the former needs Phe318 to be out of the way to the iron. Additionally, these docking solutions show different degrees of rotation of the porphyrin around the linker. This highlights a certain amount of flexibility of the porphyrin that could be related to transient interactions between the metal and the protein. To further analyze this hypothesis, classical molecular dynamics (MD) was performed.

**Figure 6:**
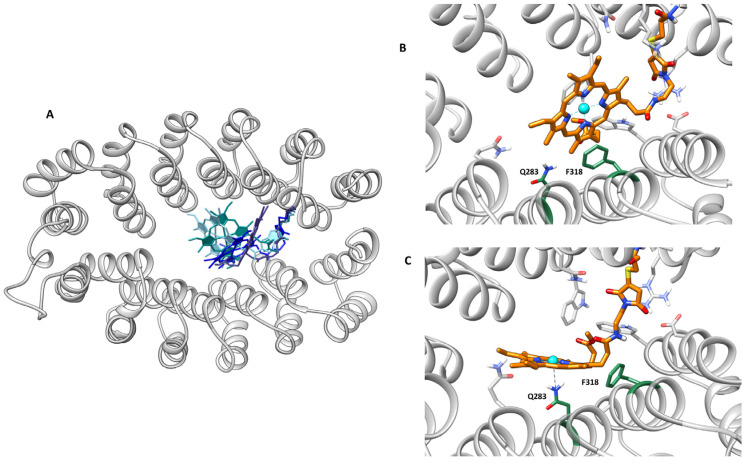
(**A**) Overlap structures of the main clusters extracted from the 400 ns MD trajectory for the (A3A3′)Y26C-**4** systems. (**B**) MD snapshot of the most populated cluster of the trajectory. (**C**) Snapshot with the closest distance between the metal and the nitrogen side chain of Gln283.

Classical MDs were carried out with the AMBER suite of programs using the AMBER20 force field [29]. The porphyrin linker was set up by defining a non-standard amino acid and by using of the MCPB.py algorithm for the parameterization of the metallic center and its first coordination sphere (see ESI for further details on the computational section) [32,33]. Calculations started from the best solution for the initial series of dockings and ran for 400 ns. The trajectory showed the stability and convergence of the system after this time (Figure S1). The calculations agreed with the flexible docking and showed a flexible cofactor in the binding site (Figure 6A). However, some orientations clearly dominated. The most populated one was similar to the best-docked structure described at the beginning of this section (Figure 6B). One can observe a very stable π-stacking between Phe318 and the porphyrin. Interestingly, the Phe318 side chain placed most of the simulation just below the metal with a position that would be occupied by an axial ligand of the metal in natural hemoenzymes. This shows that only one face of the porphyrin is accessible for catalysis and has a well-defined and asymmetric distal environment. Alternative orientations correspond to geometries in which the interaction Phe318-**4** is lost. When such an interaction is lost, compound **4** behaves very freely in the interface between the A3 and A3′ subdomains. Importantly, one of the most populated geometries of these alternative orientations showed short distances between Gln283 and the cobalt (Figure 6C), some consistent with direct Co-Gln283 coordination, and others with a bridged water molecule between the amino acid and the metal. Such interaction is only possible because the absence of the interaction Phe318-**4** is associated with an increase in the accessibility of the metal. 

In conclusion, the molecular modeling study showed that: (1) The linkage of compound **4** to (A3A3′)Y26C led to a macrocycle located at the interface between both subdomains and excluded from the solvent; (2) the main orientation of **4** in A3A3′ presented a strong contact between Phe318 and the macrocycle that aided in the packing of the porphyrin hydrophobically to one domain and led to an asymmetric environment on the distal side; and (3) that possible coordination of the metal would only appear on transient structures with Gln283. 

### 2.5. Photocatalytic Activity of the (A3A3′)Y26C-***4*** Biohybrid

The ability of the (A3A3′)Y26C-**4** biohybrid to catalyze not only the production of H_2_, but also to reduce CO_2_ under photoinduced conditions was explored. [Ru(bpy)_3_]^2+^ (bpy = 2,2′-bipyridine) was chosen as the photosensitizer and ascorbic acid as the sacrificial electron donor agent, since it has previously been shown that under irradiation at 405 nm, the excited state of [Ru(bpy)_3_]^2+^ is reduced by ascorbic acid to [Ru(bpy)_3_]^+^, which in turn transfers electrons to the catalyst, generating its active form, which is capable of reducing substrates such as protons [34] or CO_2_ [35].

#### 2.5.1. Light-Driven H^+^ Reduction

**Figure 7:**
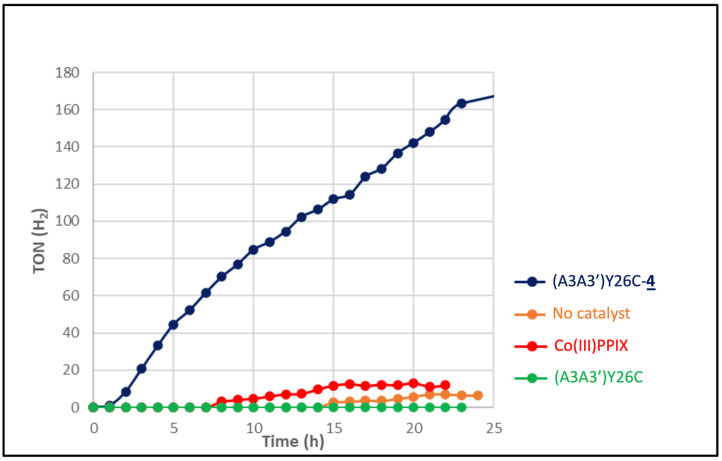
H_2_ produced over time from the photoinduced reduction of protons by (A3A3′)Y26C-**4** and CoPPIX at pH 7.0 under argon. The experiments were carried out in 100 mM ascorbic acid, 100 mM potassium phosphate buffer in the presence of 1 mM [Ru(bpy)_3_]^2+^ as a photosensitizer, and 7 μM (A3A3′)Y26C-**4** (•), Co(III)PPIX (•), (A3A3′)Y26C (•) as the catalyst or without catalyst (•) under white LED light, λ > 405 nm irradiation. All experiments were carried out at least twice.

To assay the production of H_2_ upon the photoinduced reduction of protons, experiments were carried out in argon degassed 100 mM ascorbic acid in the presence of 1 mM [Ru(bpy)_3_]^2+^ as a photosensitizer and either 7 μM (A3A3′)Y26C-**4** or 7 μM Co(III)PPIX as a catalyst in 100 mM potassium phosphate buffer at pH 7.0 under irradiation with a white LED light at λ > 405 nm. Figure 7 shows the evolution of the turnover number (TON) related to H_2_ production over time for 25 h. It appears that in the presence of the A3A3′Y26C-**4** biohybrid, the production of H_2_ increases over time to reach a value of 163 TON after 25 h. In contrast, when the metalloporphyrin CoPPIX was used as a catalyst, only a low production of H_2_ not exceeding 13 TON was observed after 25 h whereas a negligible H_2_ production was observed in the presence of the non-modified (A3A3′)Y26C protein or in the absence of a catalyst. These results clearly show that the production of H_2_ catalyzed by the cobalt(III)–porphyrin was greatly enhanced by a factor of at least 10, when the latter was buried in the hydrophobic cavity of the (A3A3′)Y26C protein. In addition, it is noteworthy that the TON value obtained with the biohybrid (A3A3′)Y26C-**4** as a catalyst was similar to that described by Ghirlanda’s group using Co-cytochrome b 562 as the catalyst [30]. It is also important to note that even after 25 h of irradiation time, the A3A3′Y26C-**4** did not show any signs of degradation and continued to show a steady production of H_2_. 

#### 2.5.2. Light-Driven CO_2_ Reduction

**Figure 8:**
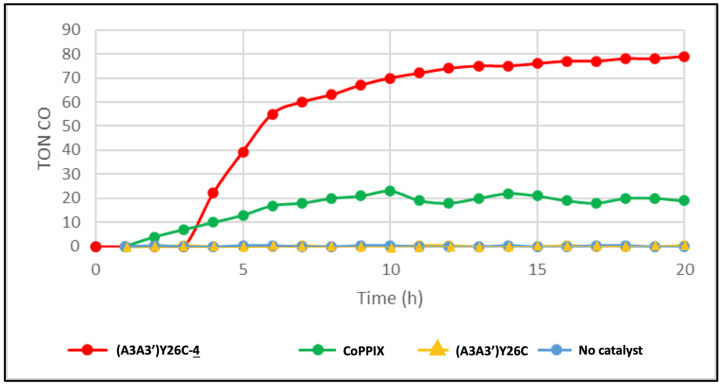
Production of CO over time from the photoinduced reduction of CO_2_ by (A3A3′)Y26C-**4** and CoPPIX at pH 7.0 in a CO_2_ saturated solution. The experiments were carried out in 100 mM ascorbic acid, 100 mM potassium phosphate saturated with CO_2_ in the presence of 1 mM [Ru(bpy)_3_]^2+^ as a photosensitizer, and 7 μM (A3A3′)Y26C-**4** (•), Co(III)PPIX (•), (A3A3′)Y26 C (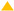) as a catalyst or without catalyst (•) under white LED light, λ > 405 nm irradiation. The experiments were carried out in duplicate.

The photoinduced reduction of CO_2_ catalyzed by (A3A3′)Y26C-**4** (7 µM) was also studied at pH 7.0 and 20 °C in a CO_2_ saturated potassium phosphate buffer containing 100 mM ascorbic acid as a sacrificial electron donor and 1 mM [Ru(bpy)_3_]^2+^ as a photosensitizer. Figure 8 shows that under irradiation with a white LED light at λ >405 nm, a significant amount of CO could be produced, reaching a plateau with a TON of 80 in about 12 h. The appearance of this plateau is probably due to the deactivation of the cobalt porphyrin through a carbonyl transfer to one of the meso positions of the porphyrin rim as reported by Jiang et al. [36]. The time lag of 3 h observed before CO production as detected by gas chromatography (GC) can probably be rationalized by an initial accumulation of the CO inside the hydrophobic pocket of the protein before being released when its concentration increases. This CO characteristic was reported to be essential for its transport from CO dehydrogenase where it is produced by the acetyl CoA synthase via direct hydrophobic gas channels before being used in a carbonylation reaction [37]. When (A3A3′)Y26C-**4** is replaced by the CoPPIX metalloporphyrin, a plateau at TON = 20 is reached after 7 h. In the control experiments, no CO production was observed in the presence of the non-modified (A3A3′)Y26C protein as a catalyst or in the absence of a catalyst. Here also, our results clearly showed that the production of CO catalyzed by the cobalt porphyrin was greatly enhanced by a factor of **4** when the latter was inserted in the hydrophobic cavity of the (A3A3′)Y26C protein. Interestingly, the TON value obtained with the biohybrid (A3A3′)Y26C-**4** as a catalyst was twice higher than that described by Ghirlanda’s group using Co-cytochrome b 562 as the catalyst (42 TON) under similar reaction conditions [18]. 

## 3. Materials and Methods

### 3.1. Synthesis of Co(III)Mal-PPIX ***4***

The Co(III)Mal-PPIX cofactor **4** was prepared in three steps.

#### 3.1.1. Protoporphyrin IX Dimethyl Ester (PPIX DME) Hydrolysis [23]

A total of 66.8 mg of protoporphyrin IX dimethyl ester PPIX DME **1** was dissolved in 6 N HCl and the solution was allowed to stand for 15 min at room temperature. This solution was then added to 200 mL chloroform overlaid with 200 mL of 10% (*w*/*v*) aqueous sodium acetate. The resulting mixture was then completely neutralized with solid NaHCO_3_ to transfer all of the porphyrin into the chloroform organic layer. This layer was then evaporated to dryness and the obtained solid was redissolved in 2 mL of 2,6 lutidine for purification via silica gel column chromatography with dichloromethane/methanol 99/1 as the eluent. PPIX monomethyl ester (PPIX MME) **2** was obtained as a purple solid (18.4 mg, 28% yield). 

^1^H NMR (CDCl_3_, 400 MHz): δ 9.85–9.98 (m, 4H, meso-H), 8.00–8.16 (m, 2H, -CH=), 6.30, 6.26, 6.15, 6.11 (s each, 4H, =CH_2_), 4.23 (m, 4H, -CH_2_-), 3.52–3.69 (m, 15H, 2, 7, 12, 18, -CH_3_, -OCH_3_), 3.23 (m, 2H, CH_2_-C_acide_), 3.14 (m, 2H, CH_2_-C_ester_), −4.14 (bs, 2H, pyrrole, NH).

MS-ESI [M + H^+^]: found. 577.278, calc. 577.280.

UV–Vis (acetonitrile) λ_max_ (nm) = 401, 503, 537, 573, 628.

#### 3.1.2. Synthesis of Maleimido-Ethylamido-Protoporphyrin IX Monomethyl Ester Mal-PPIX MME **3**

PPIX MME **2** (28.5 mg, 49.42 µmol) was dissolved in 1 mL of anhydrous DMF and 15 μL of triethylamine was added. PyBOB (36 mg, 69.18 µmol) dissolved in 1 mL anhydrous DMF was added to this solution and the reaction medium was left under stirring for 30 min at room temperature (RT). To this solution, a solution of *N*-(2-aminoethyl)malemide trifluoroacetate salt (13.8 mg, 54.36 µmol) in anhydrous DMF (1 mL) was added quickly. The resulting mixture was stirred at room temperature overnight and protected from light. The solvent was removed and the crude product was purified by silica gel chromatography (eluted with 1% MeOH in CHCl3) to give maleimido-ethylamido-protoporphyrin IX monomethyl ester Mal-PPIX MME **3** as a brown solid (15.7 mg, 45%).

^1^H NMR (CDCl_3_, 400 MHz): δ 9.89–10.12 (m, 4H, meso-H), 8.17–8.29 (m, 2H, -CH=), 6.36, 6.32, 6.18, 6.12 (s each, 4H, =CH_2_), 6.18 (br, 1H, -NH), 4.52, 4.50 (s, 2H, maleimide), 4.27–4.39 (4H, -CH_2_-), 2.97–3.03 (M, 2H, CH_2_-C_ester_) 3.23–3.27 (m, 6H, CH_2_N_maleimide_, CH_2_NH_amide_, CH_2_C_amide_), 3.52–3.66 (m, 15H, 2, 7, 12, 18, -CH_3_; -OCH_3_), −4.14 (bs, 2H, pyrrole, NH).

MS-ESI [M + H^+^] = found. 699.327, calc. 699.326.

UV–Vis (DCM), λ_max_ (nm) = 400, 503, 536, 570, 628, 662.

#### 3.1.3. Insertion of Cobalt into Maleimido-Ethylamido-Protoporphyrin IX Monomethyl Ester Mal-PPIX MME

In a 10 mL round bottom flask, 15.7 mg (22.5 µmol) of compound **3** was dissolved in 3 mL anhydrous DMF and a solution of Co(OAc)_2._ 4 H_2_O (55.96 mg, 225 µmol) in 3 mL anhydrous DMF was added dropwise. The solution was heated at 70 °C until the metalation was completed, as proven by UV–Visible spectroscopy. After the solvent was removed under reduced pressure, the residue was dissolved in 3 mL DCM. The organic layer was then washed three times with 100 mL of water, dried over Na_2_SO_4_, filtered, and the solvent was removed under reduced pressure to obtain Co(III)-Mal-PPIX MME **4** as a dark green solid (16.5 mg, 97%).

MS-ESI [M + H^+^] = found: 755.235, calc. 755.239.

UV–Vis (H_2_O), λ_Max_ (nm) = 411, 527, 561.

### 3.2. Production and Purification of αRep Proteins

The production and the purification of the αRep proteins were carried out essentially as reported in a previous paper [24].

#### 3.2.1. Protein Production

TheBL21-Gold(DE3)pLys bacteria strain was transformed with the plasmid encoding for the αRep protein and grown on 2YT-agar plates supplemented with ampicillin (100 μg/mL) and 1% glucose. One clone was picked and grown overnight at 37 °C in 2YT media supplemented with ampicillin (100 μg/mL) and 1% glucose. The overnight preculture was used to inoculate 1 L of fresh 2YT medium containing 100 μg/mL ampicillin and distributed into two 2 L flasks. Cells were grown at 37 °C until the optical density at 600 nm reached 0.8. Protein expression was then induced by the addition of 500 μM IPTG and cultures were further incubated at 37 °C for 4 h. Cells were harvested by centrifugation for 20 min at 5000× *g* at 4 °C and resuspended into 40 mL of washing buffer (50 mM sodium phosphate, 300 mM NaCl, 20 mM imidazole pH 8) supplemented with a tablet of EDTA-free anti-protease cocktail (Roche Diagnostics GmbH, Mannheim, Germany). Suspended cells were stored at −80 °C.

#### 3.2.2. Protein Purification

Frozen cells from 1 L of bacterial culture were defrosted and a tablet of EDTA-free anti-protease cocktail (Roche) and 2 μL of DNase I were added. After 30 min of incubation at 4 °C, cells were lysed by five 30 s cycles of sonication on ice and centrifuged for 1 h at 10,000× *g* at 4 °C. His-tagged proteins were purified from the crude supernatant using nickel-affinity chromatography on a 5 mL Ni-NTA column. The column was washed with 150 mL of washing buffer (50 mM sodium phosphate, 300 mM NaCl, 20 mM imidazole pH 8) and proteins were eluted in six 1 mL fractions in the elution buffer (50 mM sodium phosphate, 300 mM NaCl, 300 mM imidazole pH 8). A typical yield of about 100 mg of protein per liter of culture was obtained. Eluted fractions were analyzed by SDS-PAGE and pure fractions of proteins were pooled. αRep protein was recovered and concentrated, resulting in a 5 mL solution that was loaded on a gel filtration column (HiLoad 16/600 Superdex 75, GE Healthcare, Buc, France) equilibrated with coupling buffer (10 mM HEPES 150 mM NaCl pH 7.75). Elution fractions after gel filtration were pooled and stored at 4 °C until chemical coupling. Purity was checked by SDS-PAGE and concentrations were obtained from the absorbance at 280 nm measured on a Nanodrop spectrophotometer (Thermo Scientific, Wilmington, DE, USA) using an extinction coefficient at 280 nm of 58,900 M^−1^ cm^−1^ calculated from the protein sequence (https://web.expasy.org/protparam/ accessed on 28 October 2022).

### 3.3. Covalent Anchoring of Compound ***4*** into (A3A3′)Y26C

In a 50 mL conical tube, 6.25 mL of a 200 μM Co(III)-mal cofactor solution in DMSO was added (final concentration 50 μM) to a solution of (A3A3′)Y26C in 10 mM HEPES 150 mM NaCl pH 7.75 at a final concentration of 5 μM, in the presence of DMSO (25% in the final coupling solution) for a final volume of 25 mL. The solution was stirred for 3 days at 4 °C. The mixture was then extracted with 25 mL of 2-butanone at 0 °C to remove the excess unreacted Co(III)-mal cofactor, and the remaining solution was cooled in an ice bath. It was then centrifuged at 5000× *g* for 5 min. The pellet containing the (A3A3′)Y26C-Co(III)Mal-PPIXMME biohybrid, (A3A3′)Y26C-**4**, was resuspended in 10 mM HEPES, 150 mM NaCl, pH 8. The obtained protein solution was then loaded on a gel filtration column (HiLoad 16/600 Superdex 75, GE Healthcare), which was equilibrated with 10 mM HEPES 150 mM NaCl, pH 8, and the (A3A3′)Y26C-**4** cofactor was eluted in a single peak. The purified biohybrid was quantified from its absorbance at 280 nm, measured on a Nanodrop spectrophotometer.

### 3.4. Photocatalysis Experiments

To a degassed solution of 1 mM [Ru^II^(bpy)_3_]Cl_2_ and 100 mM NaAsc, the biohybrid protein was added to lead to a final concentration of 7 µM in a total volume of 3 mL of 0.1 M phosphate buffer, pH 7. The solution was further degassed with Ar and then irradiated with a SugarCube white LED (200 Wm^−2^; filtered at λ > 420 nm) for 25 h. During irradiation, the solution was stirred and thermostated at 20 °C with the RM6 Lauda cooling system. Gas samples from the overhead space were automatically injected into a MicroGC Fusion Gas Analyzer (Inficon, Bad Ragaz, Switzerland) every hour. For CO_2_ reduction, the cell was saturated with CO_2_ gas. All experiments were made at least in duplicate.

### 3.5. Spectroscopic Characterization of the (A3A3′)Y26C-***4*** Biohybrid

Electronic absorption spectra of the oxidized and reduced (A3A3′)Y26C-**4** biohybrid were recorded and compared to the spectra of other Co-PPIX–protein complexes previously reported in the literature. The 1 mL samples of a 7.5 μM solution of purified (A3A3′)Y26C-**4** were prepared in argon-sparged 10 mM HEPES, NaCl 150 mM buffer, pH 8.0 and placed in a septum-sealed cuvette to ensure anaerobic conditions. The reduction was initiated via the addition of 1.5 mM sodium dithionite. Spectra were recorded every five minutes at RT until the absorbance changes were no longer observed.

### 3.6. Spectroscopic Characterization of the (A3A3′)Y26C-***4*** Biohybrid

Circular dichroism (CD) spectra were recorded from 185 nm to 260 nm with a data pitch of 0.2 nm, a scan speed of 50 nm min^−1^, a response time of 0.5 s, and a bandwidth of 2 nm using quartz cells with a 1 mm path length, on a Jasco J810 dichrograph. Each spectrum was recorded five times and averaged. The CD signal was corrected with buffer subtraction, resulting in the ellipticity (θ) in mdeg. Data were acquired from a 2.2 µM sample of the biohybrid previously desalted on a Zeba spin desalting column (Thermo Scientific, Wilmington, DE, USA) equilibrated with 20 mM sodium phosphate buffer, pH 7.5.

### 3.7. Computational Methods

The coordinates from the modeled A3A3′-Y26C-MnTPP were obtained from a previous study [21]. The covalent MnTPP cofactor was removed and MD simulation was performed to obtain the most representative structure of the native form of A3A3′. Cys26 was mutated using the Dunbrack library available at UCSF Chimera [30]. The cofactor **4** structure (Figure S2) was optimized with Gaussian16 [38] via QM calculations in water solvent (SMD) [39] using B3LYP functional and Grimme’s dispersion D3 with 6-31G(d,p) for CHONS atoms and SDD + F for Co [31]. The oxidation state of Co was set to III and the spin state was singlet.

Covalent dockings were performed using GOLD5.8.0 [31], using as the score function Goldscore with parameters adapted for metals [40]. Covalent linkage was performed with a CA-Cys26 Radius search set to 15 Å and 50 runs were performed. Flexibility to certain residues was given by allowing free rotation of the side chains.

MD simulations were prepared using xleap [41], using as the force field FF19SB for proteins, TIP3P for water, and GAFF for non-standard residues. MCPB.py was used to parametrize cofactor **4** using the Seminario method [42] for force constants and parameters and RESP for charge, and the RESP method for charges [43]. Optimization, frequency, and RESP calculations were performed at the same level of theory previously specified. The system was solvated using a cubic water box and Na^+^ as counter ions were added to neutralize. MD simulations were performed using AMBER20 software [32]. Three different energy minimizations were performed to relax the system, followed by a heating step in which the system equilibrated from 0 K to 300 K. Finally, NVT and NPT equilibrations were performed, followed by a 400 ns production run. Detailed computational [29,31,32,38,39,41,42,43,44,45] can be found in the Supplementary Materials.

## 4. Conclusions

In summary, an artificial metalloenzyme was obtained by the covalent coupling of Co(III)Mal-PPIXMME **4** to the mutant (A3A3′)Y26C of an artificial bidomain protein named alpha Rep (αRep). This new artificial metalloenzyme (A3A3′)Y26C-**4** was characterized by Maldi-TOF MS, circular dichroism, and UV–Visible spectrophotometry. A comparison of the maxima of absorption of (A3A3′)Y26C-**4** with those of various Co(II)– and Co(III)–PPIX-substituted hemoproteins (Table 1) suggested the possible coordination of the cobalt ion by an axial histidine. However, investigation of the structure of this biohybrid and the positioning of the residues near the metal complex using theoretical experiments suggested the possible coordination of the metal by an axial glutamine Gln283, although it would be transient in nature and mutagenic efforts may be needed to improve axial coordination. 

The (A3A3′)Y26C-**4** biohybrid was then shown to be able to catalyze, not only the photoinduced production of H_2_, but also the reduction of CO_2_ into CO. While the Co(III)porphyrin catalyst alone showed a low activity with respect to these two reactions, more than 10 times more H_2_ and 4 times more CO_2_ were produced in the reaction, whereby the Co(III)porphyrin was buried within the hydrophobic cavity of the protein. Based on these results, we will thus be able in the future to optimize our biohybrid by carrying out different mutants for axial coordination and also to introduce positive charges on the proximal face of the biohybrid, which should increase the local concentration of CO_2_ in the enzymatic cavity to enhance its conversion into CO.

## Figures and Tables

**Table 1:** Comparison of the maxima of absorption of (A3A3′)Y26C-**4** with those of various Co(II)– and Co(III)–PPIX-substituted hemoproteins produced either in vivo or in vitro through reconstitution with analogous proteins with CoPPIX [28].

	Co(III) λ_Max_ (nm)				Co(II) λ_Max_ (nm)			
Protein	Ligands	Soret	β	α	Ligand	Soret	α/β	Ref.
Mb	**His/H_2_O**	424	535	572	**His**	406	558	[24]
MbH64VV68A	**His/H_2_O**	428	540	572	**His**	406	555	[25]
P450_Cam_	**Cys/H_2_O**	422	538	570	**Cys**	404	556	[26]
CYP119	**Cys/H_2_O**	422	539	566	**Cys**	410	558	[27]
Oxd	**His/H_2_O**	429	542	574	**His**	402	559	[28]
HRP	**His/H_2_O**	421	533	565	**His**	401	553	[24]
DYP	**His/H_2_O**	422	534	567	**His**	403	564	[28]
Cat	**His/?**	427	537	570	**His**	403	561	[28]
**(A3A3′)Y26C-4**	**?**	**423**	**535**	**569**	**?**	**399**	**562**	**This** **work**

## Data Availability

Not applicable.

## Supplementary Materials

The following supporting information can be downloaded at: https://www.mdpi.com/article/10.3390/ijms232314640/s1.
