# Supplementary material for: Photocatalytic Hydrogen Production and Carbon Dioxide Reduction Catalyzed by an Artificial Cobalt Hemoprotein
Source: Int J Mol Sci. 2022 Nov 24;23(23):14640. doi: 10.3390/ijms232314640 (PMC9736947; doi:10.3390/ijms232314640)
Supplement: Supplementary file 1 [file ijms-23-14640-s001.zip › ijms-2045580-supplementary.pdf]

Supporting Information

Photocatalytic hydrogen production and carbone dioxide reduction catalyzed by an artificial cobalt hemoprotein

Guillermo A. Oliveira Udry [1], Laura Tiessler-Sala [2], Eva Pugliese [1], Agathe Urvoas [3], Zackaria Halime [1], Jean-Didier Maréchal [2], Jean-Pierre Mahy [1,*], Rémy Ricoux [1]

[1] UMR 8182, CNRS, Institut de Chimie Moleculaire & des Matériaux d'Orsay, UniversityParis Saclay, F-91405 Orsay, France
[2] Departament de Química, Universitat Autònoma de Barcelona, 08193 Bellaterra, Spain
[3] CEA, CNRS, Institute for Integrative Biology of the Cell (I2BC), University Paris-Saclay, 91198 Gif-sur-Yvette, France
* Correspondence: jean-pierre.mahy@universite-paris-saclay.fr

1. Computational methods.

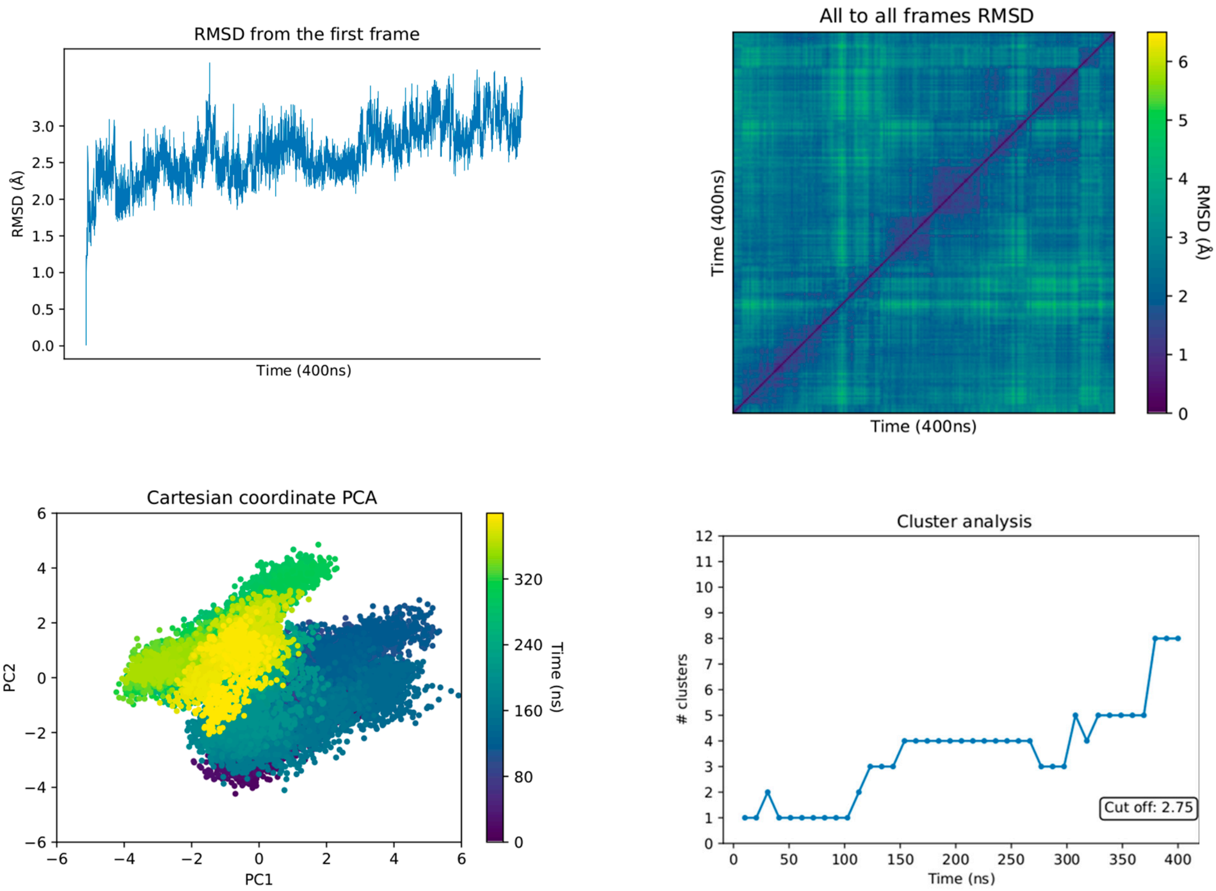

Figure S1: MD convergence analysis: RMSD, all-to-all RMSD, PCA and cluster counting.

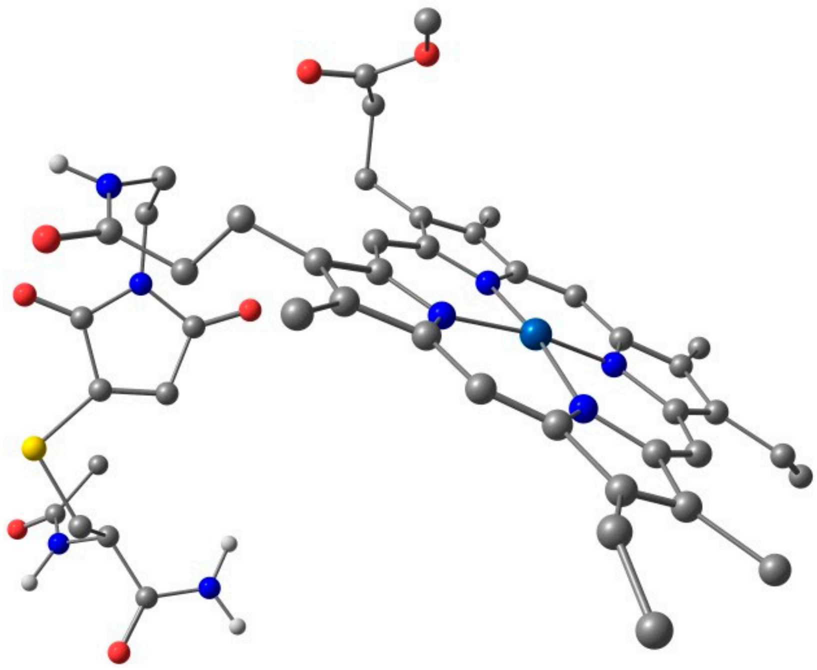

Figure S2: Optimized structure of cofactor **4** at QM level.

Detailed methods on MD simulation:

MD simulations were prepared using xleap [41], using as the force field FF19SB for proteins, TIP3P for water and GAFF for non-standard residues. MCPB.py was used to parametrize the metallic center of cofactor 4 and its first coordinating sphere [32]. Force constants and equilibrium parameters were obtained by performing optimization and frequency calculations and then MCPB.py applied the Seminario method [42]. The charges were assigned using the RESP method [43]. Optimization, frequency and RESP calculations were performed with Gaussian16 [38] via in water solvent (SMD) [39] using B3LYP functional and Grimme's dispersion D3 with 6-31G(d,p) for CHONS atoms and SDD + F for Co [31].

The system was solvated using an explicit approach cubic water box with a 10Å around the protein and 17 Na+ as counter ions were added to neutralize. MD simulations were performed using periodic boundary conditions with AMBER20 software [29]. MD simulation was performed under constant pressure with isotropic position scaling to maintain an average pressure of 1 atm. For non-bonded interactions a cut-off 8 Å was used and for long-range electrostatic interactions the PME method was applied [44]. SHAKE algorithm was applied to constrain all bonds involving hydrogen [45].

Three different energy minimizations were performed to relax the system using both steepest descent and conjugated gradient methods. In the first minimization the protein was restrained to allow water and ions to equilibrate around the protein, whereas in the second minimization only backbone was restrained to avoid side-chain clashes. In the last minimization step, there were no restrained atoms and the entire system was minimized. This was followed by a heating step in which the system equilibrated from 0 K to 300 K with restrains on the backbone. Finally, NVT and NPT equilibrations were performed, followed by a 400 ns production run.

The MD simulation trajectories convergence were analysed by a set of tools including RMSD, all-to-all RMSD, counting clustering method and PCA analysis. Furthermore, structural clustering analysis to characterize the most populated clusters were performed with cpptraj from AmberTools20 [29].

29. Case, D.A.; Belfon, K.; Ben-Shalom, I.Y.; Brozell, S.R.; Cerutti, D.S.; Cheatham, T.E.I.; Cruzeiro, V.W.D.; Darden, T.A.; Duke, R.E.; Giambasu, G.; et al. *AMBER 2020*; University of California: San Francisco, CA, USA, 2020.
31. Ehlers, A.W.; Böhme, M.; Dapprich, S.; Gobbi, A.; Höllwarth, V.; Jonas, A.; Köhler, K.F.; Stegmann, R.; Veldkamp, A.; Frenking, G. A set of f-polarization functions for pseudo-potential basis sets of the transition metals Sc/Cu, Y/Ag and La/Au. G. *Chem. Phys. Lett.* **1993**, *208*, 111–114. https://doi.org/10.1016/0009-2614(93)80086-5
32. Li, P.; Merz, K.M., Jr. MCPB.py: A Python Based Metal Center Parameter Builder. *J. Chem. Inf. Model.* **2016**, *56*, 599–604. https://doi.org/10.1021/acs.jcim.5b00674.
38. Frisch, M.J.; Trucks, G.W.; Schlegel, H.B.; Scuseria, G.E.; Robb, M.A.; Cheeseman, J.R.; Scalmani, G.; Barone, V.; Petersson, G.A.; Nakatsuji, H.; et al. *Gaussian 16, Revision C.01*; Gaussian, Inc.: Wallingford, CT, USA, 2016.
39. Marenich, A.V.; Cramer, C.J.; Truhlar, D.G. Universal Solvation Model Based on Solute Electron Density and on a Continuum Model of the Solvent Defined by the Bulk Dielectric Constant and Atomic Surface Tensions. *J. Phys. Chem. B* **2009**, *113*, 6378–6396. https://doi.org/10.1021/jp810292n

41. Cornell, W.D.; Cieplak, P.; Bayly, C.I.; Gould, I.R.; Merz, K.M.; Ferguson, D.M.; Spellmeyer, D.C.; Fox, T.; Caldwell, J.W.; Kollman, P.A. A Second-Generation Force Field for the Simulation of Proteins, Nucleic Acids, and Organic Molecules. *J. Am. Chem. Soc.* **1995**, *117*, 5179–5197. https://doi.org/10.1021/ja00124a002.
42. Seminario, J.M. Calculation of intramolecular force fields from second-derivative tensors. *Int. J. Quant. Chem.* **1996**, *60*, 1271–1277. https://doi.org/10.1002/(SICI)1097-461X(1996)60:7<1271::AID-QUA8>3.0.CO;2-W.
43. Bayly, C.I.; Cieplak, P.; Cornell, W.; Kollman, P.A. A well-behaved electrostatic potential based method using charge restraints for deriving atomic charges: The RESP model. *J. Phys. Chem.* **1993**, *97*, 10269–10280. https://doi.org/10.1021/j100142a004.
44. Ryckaert, J.P.; Ciccotti, G.; Berendsen, H.J.C. Numerical integration of the cartesian equations of motion of a system with constraints: molecular dynamics of n-alkanes. *J. Comput. Phys.* **1977**, *23*, 327–341. http://dx.doi.org/10.1016/0021-9991(77)90098-5.
45. Essmann, U.; Perera, L.; Berkowitz, M.L. A smooth particle mesh Ewald method. *J. Chem. Phys.* **1995**, *103*, 8577–8593. https://doi.org/10.1063/1.470117.
